# Early and Late Postnatal Myocardial and Vascular Changes in a Protein Restriction Rat Model of Intrauterine Growth Restriction

**DOI:** 10.1371/journal.pone.0020369

**Published:** 2011-05-31

**Authors:** Carlos Menendez-Castro, Fabian Fahlbusch, Nada Cordasic, Kerstin Amann, Kathrin Münzel, Christian Plank, Rainer Wachtveitl, Wolfgang Rascher, Karl F. Hilgers, Andrea Hartner

**Affiliations:** 1 Department of Pediatrics and Adolescent Medicine, University of Erlangen-Nürnberg, Erlangen, Germany; 2 Department of Nephropathology, University of Erlangen-Nürnberg, Erlangen, Germany; 3 Department of Nephrology and Hypertension, University of Erlangen-Nürnberg, Erlangen, Germany; Leiden University Medical Center, Netherlands

## Abstract

Intrauterine growth restriction (IUGR) is a risk factor for cardiovascular disease in later life. Early structural and functional changes in the cardiovascular system after IUGR may contribute to its pathogenesis. We tested the hypothesis that IUGR leads to primary myocardial and vascular alterations before the onset of hypertension. A rat IUGR model of maternal protein restriction during gestation was used. Dams were fed low protein (LP; casein 8.4%) or isocaloric normal protein diet (NP; casein 17.2%). The offspring was reduced to six males per litter. Immunohistochemical and real-time PCR analyses were performed in myocardial and vascular tissue of neonates and animals at day 70 of life. In the aortas of newborn IUGR rats expression of connective tissue growth factor (CTGF) was induced 3.2-fold. At day 70 of life, the expression of collagen I was increased 5.6-fold in aortas of IUGR rats. In the hearts of neonate IUGR rats, cell proliferation was more prominent compared to controls. At day 70 the expression of osteopontin was induced 7.2-fold. A 3- to 7-fold increase in the expression of the profibrotic cytokines TGF-β and CTGF as well as of microfibrillar matrix molecules was observed. The myocardial expression and deposition of collagens was more prominent in IUGR animals compared to controls at day 70. In the low-protein diet model, IUGR leads to changes in the expression patterns of profibrotic genes and discrete structural abnormalities of vessels and hearts in adolescence, but, with the exception of CTGF, not as early as at the time of birth. Invasive and non-invasive blood pressure measurements confirmed that IUGR rats were normotensive at the time point investigated and that the changes observed occurred independently of an increased blood pressure. Hence, altered matrix composition of the vascular wall and the myocardium may predispose IUGR animals to cardiovascular disease later in life.

## Introduction

Cardiovascular events like stroke or myocardial infarction are among the leading factors of morbidity and mortality in the western worId. These diseases are the consequence of atherosclerosis which in turn is often brought about by multiple risk factors.

Numerous epidemiologic and animal studies prove that intrauterine growth restriction (IUGR), which affects about 5–10% of all newborns, is an important risk factor for the development of the metabolic syndrome later in life [1]. In former IUGR individuals, type 2 diabetes, hypertension and hyperlipidemia occur more frequently than in individuals with normal birth weight [1], [2]. Two recent clinical studies by Crispi et al. [3], [4] provided evidence that in humans IUGR results in early fetal signs of cardiac dysfunction and manifest cardiovascular changes already in childhood. Moreover, a direct correlation between low birth weight and atherosclerosis later in life could be demonstrated [5]. As a consequence a higher incidence of coronary heart disease is described in former IUGR patients [6]. Studying the aetiopathogenetic interrelation between the fetal and neonatal problem of IUGR and cardiovascular diseases at adulthood, Barker and colleagues created the term of “fetal programming”. They postulated that fetal adaptation to an adverse intrauterine environment modifies cellular differentiation and tissue structure permanently and thus impairs cardiovascular structure, function and integrity [6], [7]. In this context it could be shown in animal studies, that IUGR associated intrauterine hypoxia leads to an altered myocardial vasculature which causes a reduced cardial performance and the morphological phenotype of dilated cardiomyopathy [8], [9]. Furthermore it could be shown that hypoxic intrauterine conditions lead to atherosclerosis in the offspring [10]. Martyn et al. [11] proposed an unfavourable relation of collagen to elastin in the walls of large vessels arising in early fetal development. Several studies in humans confirmed that a number of early changes in vessel walls are associated with IUGR [12]–[14].

In humans, IUGR may be brought about by different factors such as decreased placental perfusion, or maternal nutritional deficiencies. To study the pathomechanisms of IUGR and its association with diseases in later life, a number of animal models of IUGR were developed [15]. We used the low protein diet model of IUGR in the rat which is widely used [16]–[18], because it is easy to handle and highly reproducible. In the offspring of rats fed on a low protein diet during gestation, a reduced number of cardiomyocytes was detected at the time of birth [19]. Later in life, former growth restricted rats had a higher degree of myocardial interstitial fibrosis [20]. Maternal low protein diet reduced aortic wall thickness and elastin content in the offspring at 12 weeks of age [21]. However, it remained unclear, if the cardiovascular changes observed in these studies are a consequence of increased blood pressure, which is frequently observed at an older age in this model [15], [16], [18], or precede the development of hypertension.

Cardiovascular disease in human develops over decades but according to the theory of fetal programming its cornerstone is already laid in childhood. We tested the hypothesis that IUGR in the low protein diet model results in early changes in the expression of matrix components and cytokines and thus leads to alterations in the structure of the heart and blood vessels long before the onset of cardiovascular disease. A dysregulation of inflammatory and fibrotic factors is known to contribute to the development of hypertension, atherosclerosis or cardiac insufficiency. For this reason, we performed invasive and non-invasive blood pressure measurements and studied expression patterns of inflammatory and fibrotic markers in the hearts and aortas of newborn and adolescent IUGR rats of the low protein diet model using real-time PCR and immunohistochemistry.

## Materials and Methods

### Animal procedures

All procedures performed on animals were carried out in accordance with guidelines of the American Physiological Society, conform to the *Guide for the Care and Use of Laboratory Animals* published by the US National Institutes of Health (NIH Publication No. 85-23, revised 1996) and were approved by the local government authorities (Regierung von Mittelfranken, AZ # 54-2531.31-12/06). Virgin female Wistar rats (220 to 300 g) were obtained from Charles River (Sulzfeld, Germany) and were housed in a room maintained at 22±2°C, exposed to a 12 hour dark/light cycle. The animals were allowed unlimited access to standard chow (#1320, Altromin, Lage, Germany) and tap water. Ten dams were time-mated by the appearance of sperm plugs and then fed a diet containing 17.2% casein or an isocaloric diet containing 8.4% casein throughout pregnancy. Diets were obtained from Altromin (# C1000, C1003). Sodium content (0.25%) of both diets was equal. Rats delivered spontaneously and the litters were reduced to 6 male pups per dam as previously described [22] to achieve equal lactation conditions. The offspring of the mothers fed with the 17.2% protein containing diet were termed NP, the offspring of the mother with protein restriction were termed LP. Surplus male pups were used for histological and molecular evaluation of neonatal aortas and hearts. During lactation, rat mothers were fed standard chow. The offspring was nursed by their mothers until weaned at day 21 to standard chow. Rats were killed by retrograde perfusion in deep anesthesia, applying 100 mg/kg ketamine (Ketavet, Pfizer GmbH) and 5 mg/kg medetomidin (Domitor, Pfizer GmbH) intraperitoneally. The offspring used for experiments was derived from at least five litters in each group. Aortas were collected after removal of the adventitia. One part of the aortas was immediately snap frozen in liquid nitrogen for preparation of RNA. Another part was fixed in methyl carnoy solution and embedded in paraffin. Hearts were removed and a 3 mm thick most apical part of left ventricular tissue was snap frozen in liquid nitrogen for preparation of RNA. Another 3 mm of the adjacent part of left ventricular tissue was fixed in methyl carnoy solution and embedded in paraffin for immunohistochemistry. 2 µm sections of fixed tissue were cut with a Leitz microtome (Leica Instruments, Wetzlar, Germany).

### Blood pressure measurements

Blood pressure values were obtained by following methods:

Tail-cuff measurements of systolic blood pressure were performed in trained rats using a non-invasive blood pressure monitoring system (TSE Technical Scientific Equipment GmbH, Bad Homburg, Germany) one day before sacrifice. Animal contacting components were pre-warmed to 30°C and rats were positioned in the restrainer. After an acclimatization phase of 10 minutes 10 measuring cycles were performed and the average of the obtained values was documented.

Additionally in the same animals, intra-arterial blood pressure measurements were performed. 70 day old rats were anesthesized with 20 mg/kg ketamine (Ketavet, Pfizer GmbH) and 0.5 mg/kg medetomidin (Domitor, Pfizer GmbH) by intraperitoneal application. Catheters were implanted in the right femoral artery and tunneled subcutaneously. Animals were antagonized with 0.2 ml atipamezol (5 mg/ml, Antisedan, Pfizer GmbH) and woke up within 3–5 minutes. After a recovery phase of 2 hours mean arterial blood pressure was recorded by a polygraph (Hellige, Freiburg, Germany) in conscious animals for 30 minutes.

In order to confirm the data obtained from our non-invasive and invasive measurements, telemetric blood pressure measurements were performed in additional animals derived from two different litters in each group. Telemetry transmitters (TA11PA-C40, Data sciences, St. Paul, MN, USA) were implanted in 60 days old rats. After seven days of postinterventional recovery, blood pressure and heart rate were documented for one week.

### Immunohistochemistry

Vascular and ventricular paraffin sections were layered with the primary antibody, and incubated at 4°C overnight. After addition of the secondary antibody (dilution 1∶500; biotin-conjugated, goat anti-rabbit IgG or rabbit anti-mouse IgG, all from Dianova, Hamburg, Germany), the staining procedures were carried out by a peroxidase detection method as described before [23]. Each slide was counterstained with hematoxylin. As a negative control, we used equimolar concentrations of preimmune rabbit or mouse immunoglobulin G. To detect macrophages, sections were stained with a monoclonal antibody to ED-1 in a dilution of 1∶250 (Serotec, Biozol, Eching, Germany), proliferating cells were stained with a monoclonal antibody to PCNA in a dilution of 1∶50 (DAKO, Hamburg, Germany). Smooth muscle actin was detected with a monoclonal antibody (1∶50) from Serotec (Biozol). The antibody to osteopontin was used as described [23]. The primary rabbit antibody to collagen I was from Biogenesis (Poole, England) and used at a dilution of 1∶1000. The rabbit polyclonal antibody to collagen IV was used at a dilution 1∶500 (Southern Biotechnology Associates, Birmingham, AL, USA). Counting of proliferating cells and macrophages was performed after staining. In vascular tissue, all ED-1 or PCNA-positive cells per cross section were counted. In the heart, ED-1 or PCNA-positive cells were counted in five to ten myocardial views per section. Expansion of smooth muscle actin, collagen I and IV staining in the media or in the myocardium was evaluated in a Leitz Aristoplan microscope (Leica Instruments) by Metaview software (Visitron Systems, Puchheim, Germany). The stained area was expressed as percentage of the total medial area.

### Real time RT-PCR

Vascular or myocardial tissue (10 mg) was homogenized in 500 µl of RLT buffer reagent (Qiagen, Hilden, Germany) with an ultraturrax for 30 seconds, and total RNA was extracted from homogenates with RNeasy Mini columns (Qiagen) according to the standard protocol. First-strand cDNA was synthesized with TaqMan reverse transcription reagents (Applied Biosystems, Darmstadt, Germany) using random hexamers as primers. The final RNA concentration in the reaction mixture was adjusted to 0.1 ng/µL. Reactions without Multiscribe reverse transcriptase were used as negative controls for genomic DNA contamination. RT-products were diluted 1∶1 with dH_2_O before PCR procedure. PCR was performed with an ABI PRISM 7000 Sequence Detector System and SYBR Green or TaqMan reagents (Applied Biosystems) according to the manufacturers instructions. Primers used for real-time RT-PCR are listed in table 1. Primer pairs and probes were designed using Primer Express software (Perkin Elmer, Foster City, CA, USA), except for transforming growth factor-β (TGF-β) [24], collagen I [25], tissue inhibitor of metalloproteases-1 (TIMP-1) [26], osteopontin [27] and monocyte chemoattractant protein-1 (MCP-1) [28]. All samples were run in triplicates. Specific mRNA levels were calculated and normalized to 18S rRNA as a housekeeping gene with the ΔΔC_T_ method as specified by the manufacturer (http://www3.appliedbiosystems.com/cms/groups/mcb_support/documents/generaldocuments/cms_040980.pdf).

**Table 1:** List of primer pairs.

**LTBP-1** (Latent TGF- β Binding Protein)	forward 5′- CGGATCCCCCTATGATCTCA -3′reverse 5′- TGACGAGGCGGTAGCAGG -3′
**CTGF** (Connective Tissue Growth Factor)	forward 5′- TGTGCACTGCCAAAGATGGT -3′reverse 5′- GGTACACGGACCCACCGA -3′
**Fibrillin-1**	forward 5′- TGCTCTGAAAGGACCCAATGT -3′reverse 5′- CGGGACAACAGTATGCGTTATAAC -3′
**Elastin**	forward 5′- GAAAACCCCCGAAGCCCTA -3′reverse 5′- CCCCACCTTGATATCCCAGG -3′
**Collagen IV (α1)**	forward 5′- AACGAAAGGGACACGAGGA -3′reverse 5′- GGCCAGGAATACCAGGAAGT -3′
**Fibronectin**	forward 5′- TTGCAACCCACCGTGGAGTATGTG -3′reverse 5′- CTCGGTAGCCAGTGAGCTTAACAC -3′
**TIMP-2** (Tissue Inhibitor of Metalloproteases-2)	forward 5′- GCTGGACGTTGGAGGAAAGA -3′reverse 5′- GCACAATAAAGTCACAGAGGGTAAT -3′probe 5′(FAM)- TCTCCTTCCGCCTTCCCTGCAATTAGA -(TAMRA)3′
**18S**	forward 5′- TTGATTAAGTCCCTGCCCTTTGT -3′reverse 5′- CGATCCGAGGGCCTCACTA -3′probe 5′(FAM)- CGCCCGTCGCTACTACCGATTGG -(TAMRA)3′

Designed SYBR Green primer pairs and primer pairs and TaqMan probes used in this study.

### Western blot analysis

From aortas of 7 newborn control and 7 IUGR rats connective tissue was carefully removed. Aortas were homogenized in a buffer containing 50 mM Tris HCl, pH 7.5, 1% Triton X-100, 0.1% deoxycholic acid, 0.1% SDS, 150 mM NaCl, 1 mM PMSF, 1 mM sodium vanadate, 14 µg/ml aprotinin. Protein concentration was determined using a protein assay kit (Pierce, Rockford, IL, USA). Protein samples were denatured by boiling for five minutes in Laemmli sample buffer and separated on a 10% denaturing SDS-PAGE gel. After electrophoresis, the gels were electroblotted onto PVDF membranes (Hybond-P, GE Amersham, Munich, Germany), blocked with Rotiblock (Roth, Karlsruhe, Germany) for 1 hour and incubated overnight with a polyclonal antibody to CTGF (SC14939, Santa Cruz, Heidelberg, Germany) in a concentration of 1∶500. CTGF was visualized with a secondary horseradish peroxidase-conjugated anti-goat IgG antibody (Santa Cruz, 1∶50000), using the ECL system (GE Amersham). Blots were quantified using a luminescent imager (LAS-1000, Fujifilm, Berlin, Germany) and Aida 2.1 image analysis software (Raytest, Berlin, Germany). Loading of the blot was quantified by reprobing with an antibody to tubulin (Sigma, Taufkirchen, Germany, 1∶10000), which was detected with a secondary horseradish peroxidase-conjugated anti-mouse IgG antibody (GE Amersham, Munich,Germany, 1∶10000).

### Analysis of data

Animals from 6 litters per group were used when neonate tissue was investigated. Animals from 5 litters per group were used when tissue from day 70 of life was investigated. Data were weighted for litter. All data are expressed as means ± standard error of the mean. Student's t-test was used to assess the differences between the groups. Our study was designed for comparisons between IUGR and control rats but not for comparisons between neonatal and d70 animals. Hence, 2-way analysis of variance was not performed. Results were considered significant when the probability of error (p) was less than 0.05.

## Results

**Figure 1:**
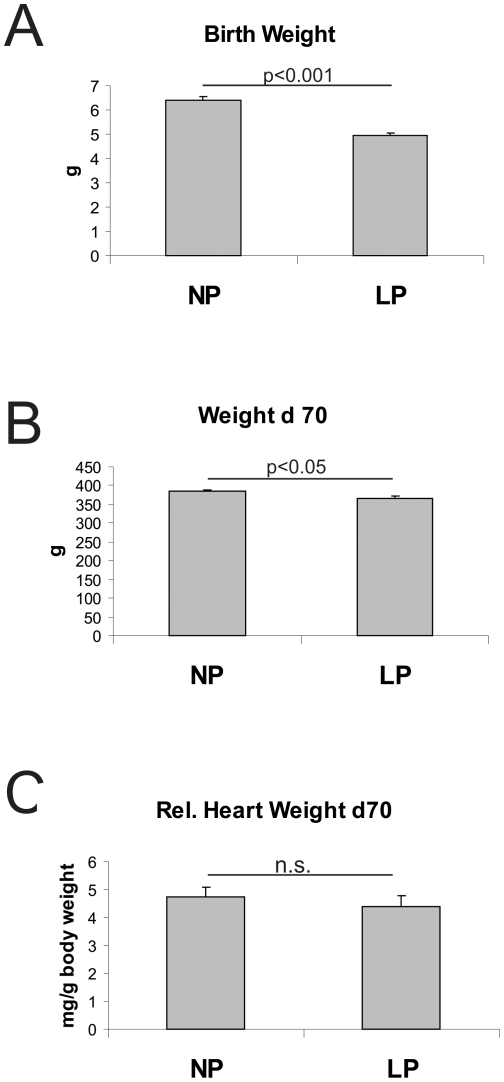
Body and relative heart weights. A: Birth weights, B: body weights at day 70 of life and C: relative heart weights at day 70 of life of rats with intrauterine growth restriction (LP) and their respective controls (NP). Data are means±sem.

**Figure 2:**
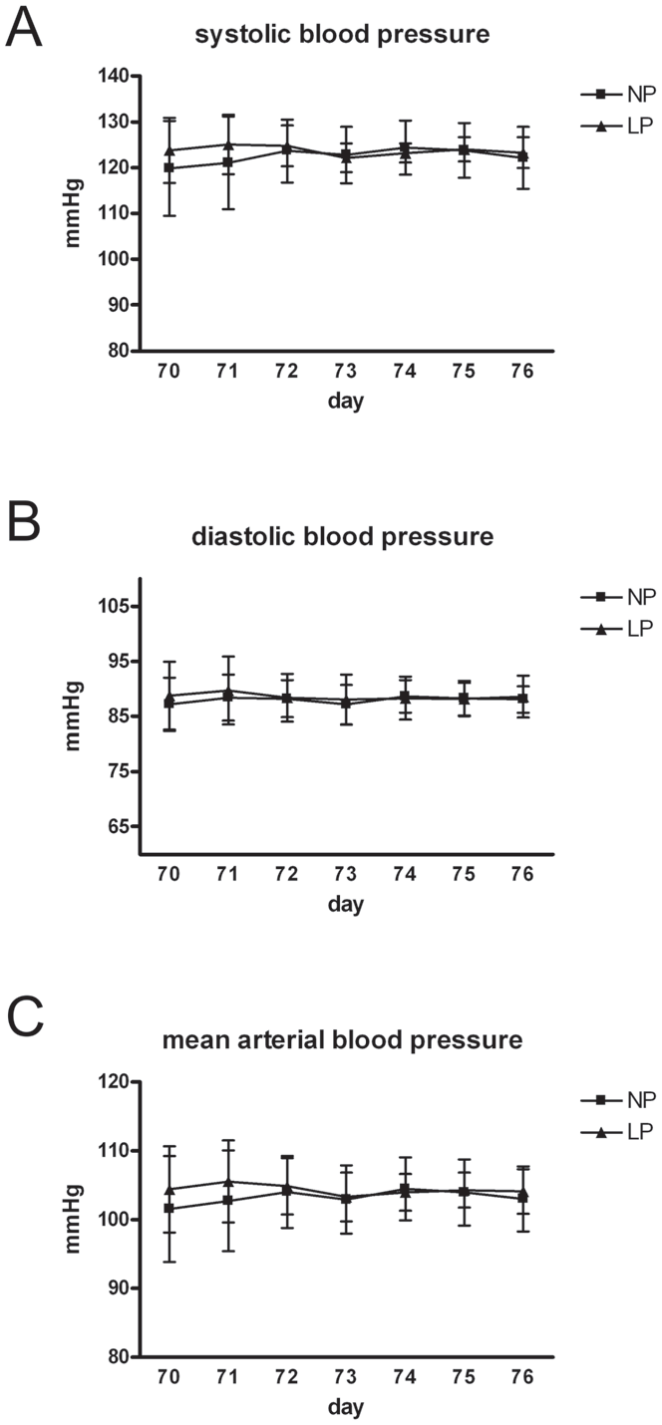
Telemetric blood pressure measurements. Systolic (A), diastolic (B) and mean arterial (C) blood pressure values of two IUGR-rats (LP) and two controls (NP) at the age of 70 days of life obtained by telemetric measurements for one week. Data are means ± sem.

Low protein diet in the pregnant dams led to a significant reduction of birth weights of their offspring (figure 1A). Some degree of catch up growth was observed in the protein restricted offspring (LP). However, at day 70 of life, body weights of LP animals were still reduced compared to the body weights of the control group (NP) (figure 1B). Relative heart weights at day 70 of life were comparable in NP and LP (figure 1C). Systolic blood pressure was comparable in the LP and NP group as assessed by tail cuff blood pressure measurements performed in 70 days old rats (134.5±7.03 mmHg in NP versus 127.3±5.03 mmHg in LP, n.s.). Moreover, intraarterial blood pressure recordings revealed no differences in mean arterial blood pressure levels of NP and LP rats at this age (105.3±4.63 mmHg in NP versus 101.3±7.12 mmHg in LP, n.s.). These findings were confirmed by telemetric blood pressure measurements in two rats of each experimental group from day 70 for one week (Figure 2).

In the aortas and hearts of neonate and 70 days old LP and NP rats, parameters of cell proliferation and differentiation (PCNA and α-smooth muscle actin), inflammation (macrophage infiltration, expression of chemotactic factors) and fibrosis (expression of profibrotic cytokines, expression and deposition of extracellular matrix molecules) were assessed.

### Cell proliferation and differentiation

In aortas of neonate LP rats, cell proliferation, as assessed by PCNA staining, occurred frequently but was not different to aortas of NP controls (table 2). At day 70 of life, cell proliferation rates in aortas were much lower compared to neonates (table 2), but again no significant differences between LP and NP rats were detected (p = 0.36; table 2). In contrast to the findings in aortas, cell growth in heart tissue seemed to be regulated by maternal protein diet: Myocardial cell proliferation rates were higher in LP than in NP neonates (table 2). In the heart tissue at day 70 of life, cell proliferation occurred less frequently than in neonate hearts, with a tendency to a reduced proliferation rate in LP compared to NP rat hearts (p = 0.08; table 2). As a vascular cell differentiation marker we investigated α-smooth muscle actin. Smooth muscle cell differentiation does not seem to be influenced by maternal protein diet: The extent of α-smooth muscle actin staining in aortas was not significantly altered in LP and NP rats at the time of birth (p = 0.06) and at day 70 of life (table 2).

**Table 2:** Markers of cell growth and differentiation.

Age	neonatal		day 70	
Diet	NP	LP	NP	LP
**Aorta:**				
Cell proliferation (% PCNA-positive cells/media cross section)	79.89±2.68	71.51±1.48	3.25±0.51	1.88±0.95
α-smooth muscle actin (% medial area)	80.29±2.72	73.41±1.84	45.74±1.94	51.74±4.67
**Heart:**				
Cell proliferation (PCNA-positive cells/myocardial view)	96.85±4.17	120.26±2.15*	9.18±0.32	7.28±0.89

Cell proliferation and areas of medial α-smooth muscle actin stain in aortas and hearts of neonatal rats and of rats at day 70 of life with intrauterine growth restriction (LP) or controls (NP).

*p<0.05 versus NP.

### Inflammation

**Figure 3:**
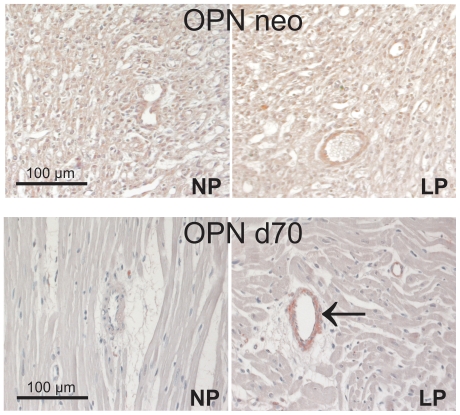
Examples of myocardial stainings for osteopontin. Representative photomicrographs of immunostainings for osteopontin (OPN) in myocardial tissue of neonatal control rats and neonatal rats with intrauterine growth restriction (neo); and in myocardial tissue of control rats and rats with intrauterine growth restriction at day 70 of life (d70). NP, control rats; LP, rats with intrauterine growth restriction. Arrow indicates positive immunoreactivity for OPN in the media of myocardial vessels in LP at day 70.

In order to study possible differences in the inflammatory response of LP and NP rats, we performed expression analyses of inflammation markers and studied macrophage infiltration in aortas and hearts of newborn rats and rats at day 70 of life. Macrophage infiltration into the aortas of newborn and 70 days old rats occurred very rarely and was comparable in LP and NP rats (table 3). In contrast, macrophage infiltration in the myocardium was reduced in the LP neonates (table 3) while at day 70 of life, myocardial macrophage infiltration was not different between NP and LP animals anymore (table 3). In the aortas of neonate rats and of rats at day 70 of life, the mRNA levels of the chemokines MCP-1 and osteopontin were below detection limits and therefore were not further evaluated. In the hearts of neonate LP rats, MCP-1 and osteopontin expression was not significantly different from NP animals (table 3). At day 70 of life, myocardial MCP-1 expression tended to be higher in LP than in NP rats. However, due to the large variability in LP rats, this increase did not reach statistical significance (p = 0.14; table 3). In contrast, the expression of osteopontin was significantly augmented in the hearts of 70 days old LP rats compared to NP rats (table 3). In order to localize the tissue compartment responsible for the increase in osteopontin expression, immunohistochemistry for osteopontin in the myocardium was performed. While in neonate heart tissue no differences in staining patterns between NP and LP rats were observed (figure 3), osteopontin immunohistochemistry in the hearts of rats at day 70 of life revealed a distinct staining of the media of myocardial vessels in LP animals which was not detected in NP animals (figure 3).

**Table 3:** Markers of inflammation.

Age	neonatal		day 70	
Diet	NP	LP	NP	LP
**Aorta:**				
Macrophage infiltration (ED-1-positive cells/cross section)	0.15±0.12	0.44±0.26	0.00±0.00	0.01±0.01
MCP-1 expression (fold induction)	0^a^	0^a^	0^a^	0^a^
Osteopontin expression (fold induction)	0^a^	0^a^	0^a^	0^a^
**Heart:**				
Macrophage infiltration (ED-1-positive cells/myocardial view)	16.01±2.23	11.25±0.90*	4.25±1.07	5.36±2.93
MCP-1 expression (fold induction)	1.00±0.14	0.70±0.34	1.00±0.54	6.11±3.07
Osteopontin expression (fold induction)	1.00±0.28	1.26±0.41	1.00±0.50	7.19±2.60*

Macrophage infiltration and expression of the chemotactic peptides MCP-1 and osteopontin in aortas and hearts of neonatal rats and in rats at day 70 of life with intrauterine growth restriction (LP) or controls (NP).

a In aortas, MCP-1 and osteopontin expression were below detection limits.

*p<0.05 versus NP.

### Fibrosis

**Figure 4:**
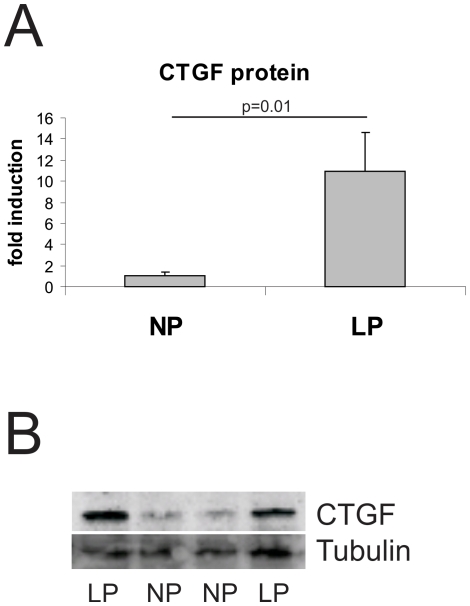
Aortal CTGF expression. A: Protein expression of connective tissue growth factor (CTGF) in aortas of newborn rats with intrauterine growth restriction (LP, n = 4) and their respective controls (NP; n = 5). Data are means ± sem. B: Example of Western blot analysis detecting connective tissue growth factor (CTGF). Tubulin was used as a loading control.

**Figure 5:**
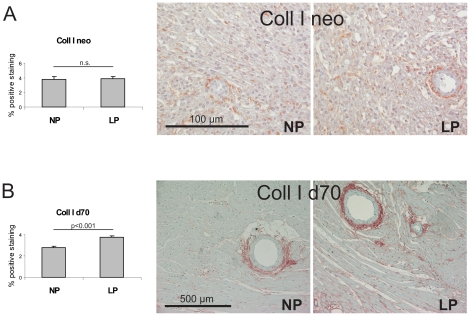
Myocardial deposition of collagen I. Evaluation of the percentage of positive immunostaining for collagen I (coll I) in myocardial tissue of (A) neonatal control rats and neonatal rats with intrauterine growth restriction (neo) with representative photomicrographs; and in myocardial tissue of (B) control rats and rats with intrauterine growth restriction at day 70 of life (d70) with representative photomicrographs. NP, control rats; LP, rats with intrauterine growth restriction. Data are means ± sem.

**Figure 6:**
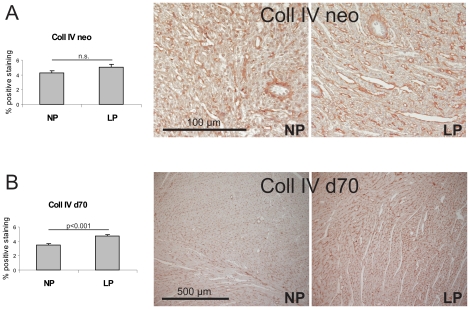
Myocardial deposition of collagen IV. Evaluation of the percentage of positive immunostaining for collagen IV (coll IV) in myocardial tissue of (A) neonatal control rats and neonatal rats with intrauterine growth restriction (neo) with representative photomicrographs; and in myocardial tissue of (B) control rats and rats with intrauterine growth restriction at day 70 of life (d70) with representative photomicrographs. NP, control rats; LP, rats with intrauterine growth restriction. Data are means ± sem.

The expression of two profibrotic cytokines, TGF-β and CTGF, was studied. While TGF-β expression was the same in neonate aortas of LP and NP rats (table 4), CTGF mRNA expression was significantly higher in neonate aortas of LP rats compared to NP rats (table 4). This was also reflected on the protein level: Western blot analysis revealed increased CTGF protein expression in neonate aortas of LP rats compared to NP rats (figure 4A+B). At day 70 of life, the aortal expression of TGF-β was still similar in both experimental groups (table 4), while due to a high variability in LP rats the expression of CTGF only revealed a tendency to be increased in LP (p = 0.06; table 4). In contrast to the findings in aortas, similar neonatal changes were not observed in myocardial tissue of LP rats: In the hearts of neonate rats, no differences in the expression levels of the profibrotic cytokines TGF-β and CTGF were detected between LP and NP (table 4). At day 70 of life, however, the myocardial expression of both TGF-β and CTGF was augmented in LP (table 4). Subsequently, we evaluated the expression of several matrix molecules. Measurements of the expression levels of the microfibrillar matrix proteins LTBP-1, fibrillin-1 and elastin did not reveal any significant differences in aortas of neonate NP and LP as well as in NP and LP at day 70 of life. In myocardial tissue of neonate NP and LP rats, the expression of microfibrillar matrix proteins did not vary significantly (p = 0.17 for LTBP; p = 0.10 for fibrillin-1 and p = 0.28 for elastin; table 4). At day 70 of life, however, the expression of microfibrillar matrix proteins was 3 to 5-fold increased in the myocardium of LP rats (figure 4). Evaluation of the expression patterns of fibronectin revealed no differences in the aorta of newborn rats, but a tendency to more aortal fibronectin expression at day 70 of life (p = 0.052; table 4). In the hearts of newborn LP rats, no increase in fibronectin expression was observed, but at day 70 of life fibronectin expression was augmented in the hearts of LP rats (table 4). Collagen I and IV expression and deposition was not different in the aortas of neonate NP and LP rats (table 4). At day 70 of life, an increase in collagen I expression and a tendency to more collagen I deposition (p = 0.07) and more collagen IV deposition was observed in LP rats (table 4). Moreover, myocardial collagen I and IV expression and deposition, although not altered in neonate LP rats, were all increased at day 70 of life (table 4, figures 5 and 6). Finally, evaluation of the aortal expression levels of two regulators of matrix turnover, TIMP-1 and TIMP-2 did not reveal any significant differences in neonate NP and LP rats nor at day 70 of life (table 4). At day 70 of life, only a tendency towards an increased expression of TIMP-2 was observed in aortas of LP animals (p = 0.053; table 4). In the hearts of LP rats, solely the expression of TIMP-2 was significantly higher than of NP rats at day 70 of life (figure 4), while the expression of TIMP-1 merely revealed a trend to be increased in the hearts of LP rats at day 70 of life (p = 0.17) due to a high variability in expression levels (table 4).

**Table 4:** Markers of fibrosis.

Age	neonatal		day 70	
Diet	NP	LP	NP	LP
**Aorta:**				
TGF-β expression (fold induction)	1.00±0.29	1.07±0.30	1.00±0.59	1.04±0.20
CTGF expression (fold induction)	1.00±0.18	3.19±0.87*	1.00±0.31	4.01±1.35
LTBP-1 expression (fold induction)	1.00±0.13	0.93±0.13	1.00±0.32	0.50±0.10
Fibrillin-1 expression (fold induction)	1.00±0.20	1.07±0.19	1.00±0.26	3.04±0.90
Elastin expression (fold induction)	1.00±0.16	0.80±0.19	1.00±0.18	1.95±0.79
Fibronectin expression (fold induction)	1.00±0.29	1.07±0.30	1.00±0.24	6.36±3.02
Collagen I expression (fold induction)	1.00±0.17	1.22±0.25	1.00±0.52	5.55±2.03*
Collagen I (% stained/medial area)	1.77±0.40	1.67±0.23	1.47±0.12	2.18±0.34
Collagen IV expression (fold induction)	1.00±0.39	1.73±0.87	1.00±0.31	1.25±0.28
Collagen IV (% stained/medial area)	3.57±0.43	2.95±0.51	8.08±0.41	10.36±0.85*
TIMP-1 expression (fold induction)	1.00±0.14	1.13±0.18	1.00±0.69	0.94±0.37
TIMP-2 expression (fold induction)	1.00±0.27	0.95±0.22	1.00±0.20	5.20±2.00
**Heart:**				
TGF-β expression (fold induction)	1.00±0.12	1.10±0.39	1.00±0.33	3.69±0.84*
CTGF expression (fold induction)	1.00±0.24	0.44±0.16	1.00±0.32	5.47±0.92*
LTBP-1 expression (fold induction)	1.00±0.17	0.66±0.15	1.00±0.40	3.59±0.63*
Fibrillin-1 expression (fold induction)	1.00±0.16	0.62±0.14	1.00±0.449	4.55±0.65*
Elastin expression (fold induction)	1.00±0.53	0.34±0.12	1.00±0.43	5.70±1.35*
Fibronectin expression (fold induction)	1.00±0.28	0.81±0.30	1.00±0.46	7.12±1.53*
Collagen I expression (fold induction)	1.00±0.35	0.84±0.34	1.00±0.55	6.38±1.32*
Collagen IV expression (fold induction)	1.00±0.08	0.91±0.14	1.00±0.21	4.07±0.22*
TIMP-1 expression (fold induction)	1.00±0.07	0.90±0.23	1.00±0.56	4.25±2.07
TIMP-2 expression (fold induction)	1.00±0.11	0.76±0.22	1.00±0.43	7.38±1.58*

mRNA expression of markers of fibrosis and type I and IV collagen stain in aortas and hearts of neonatal rats and in rats at day 70 of life with intrauterine growth restriction (LP) or controls (NP).

*p<0.05 versus NP.

## Discussion

The salient finding of our study is the presence of changes in the expression patterns of profibrotic genes, and of discrete structural abnormalities of vessels and hearts in adolescence, in the low protein diet model of IUGR despite normal blood pressure at the time point investigated. IUGR is often associated with hypertension later in life [17], [29], which might lead to cardiovascular changes similar to those observed. In our animal model of IUGR, however, blood pressure was not yet increased in IUGR at the time points we studied. Thus, it seems conceivable that changes in aortal and myocardial tissue in our study are not the secondary result of hypertension, but the potential primary precursors of cardiovascular disease following IUGR.

IUGR is considered as the consequence of a disturbed intrauterine environment. Therefore, a variety of animal models exist to induce IUGR e.g. maternal protein restriction [30], maternal exposure to hypoxia [31] or the arteriae uterinae ligation model [32]. These models may to some extent reflect the different factors which can bring about IUGR in humans in different clinical situations, such as nutritional deficiencies or placental hypoperfusion, respectively [33]. For example, the hypoxic models lead to hypoxia of the placenta and the fetus without impairing the maternal metabolic and endocrinologic status [33]. On the other hand, surgical models best represent placental hypoperfusion but may entail unwanted side effects by the necessary operative intervention. Even a sham operation at this stage of pregnancy, or the necessary anaesthesia, may affect the growth of the fetus [34].

Our findings are limited to the protein restriction model in rats. Protein restriction is presumably not a major factor causing IUGR in developed countries. However, the effects of maternal protein restriction on the fetus are not limited to an isolated nutritional defect. Data from various animal studies suggest that maternal protein restriction affects placental size, one of the major indicators of placental insufficiency [35], and alters placental vasculature and the exchange barrier [36], [37]. Despite its limitations, the animal model of maternal protein restriction used in this study thus comprises both fetal hypoxia and nutrient restriction. On the other hand one has also to face the disadvantages of this animal model: The outcome varies between the species and the composition of the nutrition used [2]. Other central aspects like the timing and the duration of protein restriction as well as maternal metabolic status have to be standardized to obtain reproducible results. The absence of hypertension is both a strength and a limitation of our study. We presume that the vascular and myocardial changes observed by us would be further aggravated by hypertension but we did not test this hypothesis. Hypertension may be present early in other models of IUGR such as uterine ligation [38] and may occur later in life in the protein restriction model [39]. We did rule out hypertension in the time period investigated, in agreement with a recent study [40], reporting radiotelemetric blood pressure results from slightly older IUGR animals in the protein restriction model.

Our study was designed for comparisons between IUGR and control rats but not for direct comparisons between neonatal and d70 animals which deserves mention as a further limitation. For example, animals from the d70 time point were from different litters than animals for the neonatal time point, and the molecular expression data for the different time points were not assayed together.

We detected an increased expression of connective tissue growth factor (CTGF) in aortas of IUGR neonates and an increased vascular expression and deposition of collagens in former IUGR rats at day 70 of life. CTGF is a growth factor involved in fibrotic changes in several organs, including blood vessels [41]. As CTGF is overexpressed in atherosclerotic lesions of rodents [42] and humans [43] and is undetectable in normal human blood vessels [43], it is thought to contribute to atherogenesis. CTGF can stimulate the production of matrix molecules, like collagen I and fibronectin [44], and induce mononuclear cell chemotaxis [45] as well as vascular smooth muscle cell growth and migration [46]. One might speculate that the early expression of CTGF in the aorta in IUGR neonates could consequently lead to the observed vascular overexpression of collagens later in life. If CTGF can also induce the expression of fibrillin-1, however, is unknown. CTGF is also regarded as a central factor regulating angiogenesis [47] and myocardial development [48]. However, in the neonatal heart of IUGR rats, no myocardial CTGF upregulation was observed, indicating that the observed aortic CTGF upregulation is a structure specific IUGR-induced change.

Although no gross alterations in aortic histology were observed, a consistent high expression of matrix molecules might result in more overt vascular changes later in life. In humans, the aortic wall of IUGR newborns was found to be thicker than in newborns with normal birth weight, although there was an extensive overlap [14]. Moreover, alterations in the composition of the vascular extracellular matrix could possibly reduce the elastic properties of the vessels and thus reduce vascular compliance, which is known to be a marker of cardiovascular disease. A shift in the relative amounts of collagen and elastin was already suggested by Martyn et al. [11] for human fetal aortas of individuals with low birth weight. This notion is supported by our data on changes in collagen expression levels while we could not show significant alterations in the expression of elastin in aortas of IUGR neonatal rats nor at day 70 of life.

In the hearts of neonatal IUGR rats, no profibrotic changes were observed in our study. However, myocardial cell proliferation was augmented in neonate IUGR rats. In contrast, Corstius et al. demonstrated a reduced number of cardiomyocytes in the hearts of neonate female rats in response to low protein diet [19]. Four weeks later, however, the number of cardiomyocytes was not different anymore in this model, as shown by the same group [49]. Thus, the augmented myocardial cell proliferation in neonate rats after IUGR described in our study might indicate a compensatory postnatal hyperplasia following an initially reduced number of cardiomyocytes. But this early myocardial remodelling might also trigger the development of fibrotic alterations later in life. In this context Lim et al. could show that former IUGR animals developed increased interstitial fibrosis of the left ventricle, suggesting that an early dysregulation of cardiomyocyte proliferation and subsequent cellular hypertrophy might result in increased accumulation of collagens at a higher age [20]. However, in this study it remained unclear, if the higher incidence of fibrosis in the left ventricle could also be a consequence of an increased blood pressure. In accordance to this study and to others in different animal models of IUGR [50], [51] we detected an increased expression and accumulation of collagens in the hearts of former IUGR individuals later in life.

Furthermore we could show an overexpression of all matrix molecules investigated, even of those known to serve elastic properties like elastin or fibrillin-1. The functional consequences of an overexpression of these molecules in heart tissue are not known [52], but Bouzeghrane et al. observed an enhanced deposition of fibrillin-1 in the fibrotic hearts of rats suffering from hypertensive disease [53], arguing for a role for fibrillin-1 in myocardial fibrosis. This could also be accelerated by higher expression levels of tissue inhibitor of metalloprotease 2 (TIMP-2), which was seen at day 70 of life in our study. TIMP-2 serves as a potent inhibitor of matrix degradation [54].

There is growing evidence for an altered immune response in IUGR individuals [22], [33]. Surprisingly, macrophage infiltration into the myocardium was lower in newborn IUGR rats than in control rats in our study, while at day 70 of life former IUGR rats tended to exhibit increased macrophage infiltration. This went along with an increased expression of the chemotactic protein osteopontin (OPN). OPN is a multifunctional molecule which also regulates macrophage associated inflammatory pathways [55]. Clinical studies proved an increased myocardial expression of OPN in acute as well as chronic heart disease, like myocardial infarction [56] or dilated cardiomyopathy [57]. Furthermore there is growing evidence that OPN is a valid predictor for the outcome of patients with chronic heart disease [58] and contributes to fibrotic myocardial remodeling [59].

Taken together, our data support the hypothesis that subtle but defined molecular and structural changes develop in IUGR before the manifestation of cardiovascular risk factors like arterial hypertension, at least in our rat model of protein restriction. These alterations may in turn predispose to cardiovascular disease later in life by increasing the vascular and cardiac vulnerability to additional stressors or risk factors.
